# An evidence-based self-management package for urinary incontinence in older women: a mixed methods feasibility study

**DOI:** 10.1186/s12894-020-00603-8

**Published:** 2020-04-20

**Authors:** Yu Fu, E. Andrea Nelson, Linda McGowan

**Affiliations:** 1 Leeds Institute of Health Sciences, University of Leeds, Leeds, LS2 9NL UK; 2 School of Health and Life Sciences, Glasgow Caledonian University, Glasgow, UK; 3 School of Healthcare, University of Leeds, Leeds, UK

**Keywords:** Urinary incontinence, Self-management, Feasibility, Randomised controlled trial, Acceptability, Mixed methods

## Abstract

**Background:**

Urinary incontinence (UI) is a distressing condition that limits women’s quality of life and places a heavy burden on health care services. Behavioural treatments are recommended as a first-line treatment. An evidence-based self-management package was developed following the Medical Research Council (MRC) framework for complex interventions. This study aimed to evaluate the feasibility and acceptability of the intervention.

**Methods:**

A mixed-methods approach was undertaken, namely a randomised controlled feasibility study with nested qualitative study. Fifty women aged 55 or over living with UI, recruited from community centres were randomly assigned to either a 3-month course with the package with a support session or a control group to receive the same package only 3 months later. Principal outcome measures were: self-reported quality of life, UI severity, self-efficacy and psychological status. Analysis of covariance was undertaken to estimate within- and between- group changes for all outcomes. Acceptability was explored using individual interviews at follow-up.

**Results:**

Fifty women were randomised (24 to intervention, 26 to control); mean age of 69.7 (±9.1) years and mean UI frequency 2.2 (±2.2) episodes/day at baseline. Overall, 49 women (98%) completed 3-month follow-up (24 in the intervention, 25 in the control). A positive trend was detected in the impact of UI on their personal relationships (− 3.89, *p* = 0.088), symptom severity (− 1.77, *p* = 0.025), UI symptoms scale (− 1.87, *p* = 0.031) and anxiety status (− 2.31, *p* = 0.001), respectively. Changes in quality of life and self-efficacy did not differ significantly between groups. Majority of women (71%) in the intervention group reported subjective improvement after 3 months. Spearman correlation coefficient was 0.43 (*p* < 0.05) between their subjective perception of change and self-efficacy. Women perceived the package being acceptable and described that the package had the potential to increase their knowledge and confidence to manage symptoms and improve quality of life.

**Conclusions:**

The study demonstrated that the self-management package is feasible and acceptable for older women with UI. Further studies are needed with a large sample size in clinical settings to evaluate the effectiveness of this package.

**Trial registration:**

ISRCTN17194896. Registered on 11th September 2019 (retrospectively registered).

## Background

Urinary incontinence (UI) is a distressing condition that limits people’s quality of life and places a burden on those affected and health and social care services [1, 2]. Prevalence in women is estimated to be around about 40%, with an increase with age [3]. Women living with UI often experience functional limitations and social embarrassment, negatively impacting their mental health [4]. Although several options are available for treating and managing UI, behavioural treatments are recommended as a first-line treatment therapies for stress, urgency and mixed UI by international UI guidelines developed worldwide [5], prior to considering more intensive treatments.

Behavioural treatment programmes focusing on single elements of behavioural strategies have been extensively researched, e.g. pelvic floor muscle exercises (PFME) [6]. These programmes are sometimes challenging to deliver as they need multiple appointments, the involvement of specialised practitioners, and may lack flexibility to respond to individual needs associated with comorbidities in older women. Many women therefore choose to disengage with the service. Only a third of women with UI consult a doctor in European countries such as France, Germany, Spain and the UK, and 20 to 25% of those experiencing significant clinical symptoms seek care and less than half of them receive treatment [7]. A multifaceted intervention involving behavioural strategies may be more effective than a single component for the management of UI in older women [8, 9].

Self-management for chronic conditions is multidimensional and defined as an intervention designed to develop individuals’ knowledge, skills or psychological and social resources and their ability to manage their health condition and consequences, through education, training and support [10]. Many self-management interventions have been developed to support people to cope with their health conditions and improve quality of life. Positive outcomes reported include a higher degree of self-efficacy, reduced physical interference, and improved mental health status at follow-up [11–21]. Although evidence for self-management programmes incorporating multifaceted behavioural treatments for use in UI among older women is currently limited, our systematic review concluded that multifaceted self-management interventions including PFME, bladder retraining, or combination behavioural techniques are beneficial.

Following the Medical Research Council (MRC) framework for developing and evaluating complex interventions [22], a self-management package was co-developed with older women with UI and health professionals providing treatment and care. The initial draft of the intervention was informed by synthesis of data from a systematic review and stakeholder interviews with women and health professionals. Both groups preferred an evidence-based self-management package that had the capacity to meet women’s individual needs with the flexibility to modify behaviours in coping with UI without significant service provision and/or intensive interaction with health professionals. The initial draft was reviewed and discussed in detail with an expert group which consisted of four women with UI, five health professionals and two lay members. Consensus was reached on the content and format of this self-management package for older women with UI using a normal group technique [23]. However, the feasibility and acceptability of the intervention remained unknown.

## Methods

### Aim

The aim of this study was to evaluate the feasibility, acceptability, and preliminary outcomes of an evidence-based self-management package for UI in older women. Specifically, it was to 1) assess the feasibility of the intervention to evaluate the effectiveness of a self-management package; 2) assess the variation of the main outcome measures to inform sample size considerations for a full randomised controlled trial (RCT); 3) provide preliminary data on the effect of the intervention; 4) understand the benefits/limitations and acceptability of the self-management package compared to no active treatment.

### Design

A mixed-methods approach comprising a two-arm RCT feasibility study with a nested qualitative study was undertaken. The qualitative study was conducted to understand how the intervention might work and explore facilitators and barriers to acceptance of the intervention [24]. This study presented adheres to CONSORT guidelines.

### Patient and public engagement

A project advisory group comprising three older women (aged 55 plus) living with UI and one nurse working in a community continence clinic was set up prior to the commencement of this study, to ensure this study addressed issues that were important and relevant to women. The meeting was led by YF and facilitated by LMc/EAN every 6 months. Participants were provided with background information and clinical guidelines for UI management and consulted for their current experiences and expectations of managing UI. They also reviewed the findings of the systematic review and interview transcripts. All highlighted the need for evidence-based practice that has the ability to engage the wider population and indicated a willingness to use it for UI self-management. The group also supported the application of ethics approval and development of interview schedules.

### Setting

This is a single-centre, randomised controlled feasibility study of self-management package versus wait-list control with 12- week post-intervention follow up. Participants were recruited using flyers posted in community centres of a local Forum for older people in West Yorkshire, UK. A short presentation was also given by the project researcher in most centres.

### Participants

Women were eligible if they were aged 55 or over, had self-reported symptom of involuntary leakage of urine and were able to read and speak English. Individuals who self-reported their UI were caused by neurological diseases affecting the brain and spinal cord, or were cognitively impaired were excluded.

A consecutive sampling strategy was applied to recruit participants in this study. Assuming an attrition rate of 20%, a target of 25 per arm was sufficient to have 20 participants in each arm by the end of the study. This has been recommended as acceptable for a feasibility trial assuming at worst a medium effect size (Cohen’s d = 0.2) for a continuous outcome and 80% power [25].

### Randomisation

All participants were recruited prior to randomisation to the intervention. Eligible women were randomised using a 1:1 ratio to either the intervention or control group. The randomisation procedure was performed by a web-based randomisation service (https://sealedenvelope.com/). Due to the nature of the intervention, this study is considered open-label and is not allocation concealed because the researcher delivering the interventions and the participants were aware of group allocation at the time of implementation.

### Intervention

The experimental intervention was the self-management package, co-developed with older women living with UI, health professionals and lay members. The aim of the package was to provide information and practical skills for women to self-manage their UI and other symptoms. Following elements were included: recognition and awareness, getting the support you need, understanding the cause, learning to manage your UI, developing a self-management plan and how can you find out more. Descriptions of self-management techniques such as PFME, bladder training and lifestyle interventions were also provided. The researcher acted as a facilitator and delivered the intervention in person immediately after the completion of baseline data collection. A self-management brochure was also distributed. The intervention group could request a single one-hour support session with the researcher if they had any difficulties in using the package.

The control group did not receive the self-management package or the support session. However they had been given a copy of the same package upon the completion of their follow-up data collection (at 3 months).

### Outcome measures

As this was a feasibility study it was appropriate to explore a range of outcome measures. Standardised self-reported measures were used to assess participants’ generic and disease-specific quality of life, UI severity, self-efficacy and psychological health respectively: the EuroQol (EQ-5D-5 L) [26], King’s Health Questionnaire (KHQ) [27], International Consultation on Incontinence Questionnaire – urinary incontinence short form (ICIQ-UI SF) [28, 29], Geriatric Self-Efficacy index for urinary incontinence (GSE-UI) [30], and Hospital Anxiety and Depression Scale (HADS) [31]. Data were collected at baseline and three-month follow-up. Patient Global Impression: Improvement (PGI-I) was obtained from the intervention group only at three-month follow up [32]. These measures have been commonly used in research and practice for women with UI.

### Data analysis

Descriptive analyses were undertaken to establish recruitment, drop-out rates and the distribution of baseline and follow-up characteristics and outcomes. For each of the above measures, analysis of covariance (ANCOVA) was conducted adjusting for their baseline values to take account of outcome imbalance at baseline and estimate the impact of intervention compared with control [33, 34]. T-tests and Chi-square tests were applied to compare two groups at baseline and follow-up for continuous and categorical variables, respectively. Intention-to-treat analysis was performed for this study. Data from all subjects were included in the analysis as randomised. To test the appropriateness of the analysis, complete case analysis was undertaken for each of the above outcome measures to identify the relative treatment effects using a linear (for continuous variables) and logistic (for binary outcomes) mixed model. For all analyses, a two-tailed *p*-value less than 0.05 was considered statistically significant and a p-value between 0.05 and 0.1 was interpreted as indicating a trend [35, 36].

### Nested qualitative study

Women in the intervention group were eligible to participate in this subsequent qualitative study aiming to understand the acceptability of the package. They were purposively recruited considering different types of UI, number of years living with UI, to enable wider discussions on the self-management package and their experience. The concept of data saturation guided sample size for this qualitative study [37].

Semi-structured individual interviews using open-ended questions were undertaken at follow-up. Interviews were conducted either face-to-face in the participants’ homes, in a meeting room at the University, or by telephone, based on participants’ preferences. Interviews were digitally recorded with permission and transcribed verbatim for analysis. Interviews focused on exploring participants’ experience of managing symptoms guided by the intervention, facilitators and barriers to the use of the package, comments on the content and format, feedback on outcome measures used and suggestions on dissemination strategy.

Data analysis was commenced during the interview phase and the transcription, and continued during the analysis of the transcriptions, hence early commencement of analysis facilitated the development of subsequent interviews [38]. Data were analysed using thematic analysis [39], involving a six-step procedure: familiarising the data, generating initial codes, searching for themes, reviewing themes, defining and naming themes, and producing the report. Although they were presented as a step-by-step procedure, the analysis was an iterative and reflexive process to finalise the themes. To ensure trustworthiness and rigour of the analysis, we double coded the data as a validity check and explored alternative interpretations of the data and through discussion with members of the research team. Interview transcripts were managed and analysed using Nvivo, a qualitative software programme for organising and coding the data [40].

## Results

### Participants characteristics

Overall 50 participants (*M*_*age*_ =69.7, *SD* = 9.1), were randomly allocated to either intervention (*n* = 24, *M*_*age*_ =69.5, *SD* = 8.9) or control group (*n* = 26, *M*_*age*_ =69.8, *SD* = 9.5). During the 3 months follow-up, no one in the intervention group requested the support session with the researcher. One woman in the control group dropped out due to admission to the hospital for other health conditions. A total of 49 participants with a mean age 69.6 (SD = 9.1) were included in the complete case analysis. No significant differences were found for all demographics and UI characteristics collected at baseline, indicating that randomisation was achieved between the intervention and control groups. The majority were white British (77.6%), completed secondary and higher education (91.8%), were retired (71.4%), and had delivered 2 or more children (71.5%). The number of participants was similar across different UI types. Please see participant’s characteristics in Table 1.

**Table 1:** Baseline demographics and characteristics

Variable	Overall		Intervention group		Control group		*p*-value
	*n*	%	*n*	%	*n*	%	
Total	49		24		25		
Age (Mean, SD)	69.7	9.1	69.5	8.9	69.6	9.6	0.994
Ethnicity							0.157
White British	38	77.6%	16	66.7%	22	88.0%	
Asian Indian	9	18.4%	7	29.2%	2	8.0%	
Mixed White and Black African	2	4.1%	1	4.2%	1	4.0%	
Education							0.486
Primary	4	8.2%	3	12.5%	1	4.0%	
Secondary	16	32.7%	9	37.5%	7	28.0%	
Further	14	28.6%	5	20.8%	9	36.0%	
Higher	15	30.6%	7	29.2%	8	32.0%	
Religion							0.108
No religion	9	18.4%	5	20.8%	4	16.0%	
Christian	31	63.3%	12	50.0%	19	76.0%	
Hindu	9	18.4%	7	29.2%	2	8.0%	
Marital status							0.365
Single	6	12.2%	3	12.5%	3	12.0%	
Married	27	55.1%	15	62.5%	12	48.0%	
Separated	1	2.0%	0	0.0%	1	4.0%	
Divorced	3	6.1%	0	0.0%	3	12.0%	
Widowed	12	24.5%	6	25.0%	6	24.0%	
Employment							0.573
Full time employed	3	6.1%	1	4.2%	2	8.0%	
Part time employed	5	10.2%	3	12.5%	2	8.0%	
Self employed	1	2.0%	0	0.0%	1	4.0%	
Not in paid employment	2	4.1%	1	4.2%	1	4.0%	
Unemployed-looking for work	1	2.0%	0	0.0%	1	4.0%	
Retired	35	71.4%	19	79.2%	16	64.0%	
Unable to work	2	4.1%	0	0.0%	2	8.0%	
Children							0.421
None	11	22.4%	5	20.8%	6	24.0%	
1	3	6.1%	1	4.2%	2	8.0%	
2	18	36.7%	9	37.5%	9	36.0%	
3	11	22.4%	4	16.7%	7	28.0%	
UI type							0.672
Stress UI	14	28.6%	8	33.3%	6	24.0%	
Urge UI	17	34.7%	7	29.2%	10	40.0%	
Mixed UI	18	36.7%	9	37.5%	9	36.0%	
Frequency of UI (average per day)							0.070
None	16	32.7%	12	50.0%	4	16.0%	
1	8	16.3%	3	12.5%	5	20.0%	
2	5	10.2%	1	4.2%	4	16.0%	
3	9	18.4%	5	20.8%	4	16.0%	
4+	11	22.4%	3	12.5%	8	32.0%	

*UI* urinary incontinence

### Outcome measures

Medians, interquartile range (IQR), mean, standard deviation (SD) and *p*-values of all baseline and follow-up outcomes are shown in Tables 2 and 3, respectively. There were no differences shown in general quality of life, UI symptoms scale and emotional health status at baseline, however groups differed with regard to KHQ severity measures (34.03 vs 48.67, *p* = 0.028) and self-efficacy (84.96 vs 57.76, *p* < 0.001). At follow-up, differences were detected in KHQ symptom severity (6.38 vs 9.04, *p* = 0.006), UI symptoms scale (5.38 vs 7.76, *p* = 0.022), self-efficacy (89.13 vs 69.08, *p* = 0.007), and anxiety (5.13 vs 7.80, *p* = 0.049) at 3-month follow-up.

**Table 2:** Median, interquartile range (IQR), mean, standard deviation (SD) and *p*-values of outcome measurements at baseline

Variable	Dimensions	Intervention group						Control group						*p*-value
		*n*	%	Median	IQR	Mean	SD	*n*	%	Median	IQR	Mean	SD	
EQ-5D-5L	Mobility													0.675
	No/slight problem	18	75.0%					20	80.0%					
	Moderate/Severe/extreme problem	6	25.0%					5	20.0%					
	Selfcare													0.527
	No/slight problem	22	91.7%					24	96.0%					
	Moderate/Severe/extreme problem	2	8.3%					1	4.0%					
	Usual activities													0.662
	No/slight problem	19	79.2%					21	84.0%					
	Moderate/Severe/extreme problem	5	20.8%					4	16.0%					
	Pain & discomfort													0.315
	No/slight problem	14	58.3%					18	72.0%					
	Moderate/Severe/extreme problem	10	41.7%					7	28.0%					
	Anxiety & depression													0.524
	No/slight problem	20	83.3%					19	76.0%					
	Moderate/Severe/extreme problem	4	16.7%					6	24.0%					
	Index	24		0.90	0.23	0.82	0.17	25		0.83	0.14	0.79	0.19	0.643
	VAS	24		77.50	27.50	71.67	18.04	25		70.00	25.00	64.40	25.59	0.258
KHQ	Part I													
	General health perception (0-100)	24		25.00	25.00	33.33	21.70	25		25.00	25.00	32.00	15.34	0.804
	Incontinence impact (0-100)	24		33.33	33.33	45.83	23.70	24		66.67	33.33	56.94	20.80	0.091
	Part II													
	Role limitations (0-100)	24		16.67	33.33	20.14	22.51	25		33.33	33.33	33.33	24.06	0.054
	Physical limitations (0-100)	24		16.67	41.67	22.92	22.42	25		33.33	33.33	30.67	20.79	0.216
	Social limitations (0-100)	24		0.00	13.89	9.03	13.19	25		0.00	22.22	16.44	24.40	0.195
	Personal relationships (0-100)	15		0.00	0.00	5.56	12.06	14		0.00	0.00	11.90	25.68	0.396
	Emotions (0-100)	24		11.11	22.22	16.67	19.93	24		11.11	11.11	17.59	14.98	0.856
	Sleep/energy (0-100)	24		33.33	41.67	37.50	28.34	25		50.00	50.00	47.33	32.16	0.263
	Severity measures (0-100)	24		33.33	29.17	34.03	22.10	25		41.67	33.33	48.67	22.91	**0.028**
	Part III													
	Symptom severity scale (0-30)	24		7.50	5.50	8.00	4.39	25		10.00	3.00	9.92	4.06	0.119
ICIQ-UI SF	Scale (0-21)	24		8.00	6.50	8.63	4.03	25		9.00	6.00	9.68	4.19	0.374
GSE-UI	Scale (0-120)	24		84.50	33.50	84.96	18.89	25		55.00	23.00	57.76	23.55	**0.000**
HADS	Anxiety scale (0-21)	24		6.50	6.50	7.13	5.12	25		7.00	5.00	7.56	4.56	0.755
	Normal	13	54.2%					13	52.0%					0.868
	Mild	7	29.2%					6	24.0%					
	Moderate	2	8.3%					4	16.0%					
	Severe	2	8.3%					2	8.0%					
	Depression scale (0-21)	24		3.00	3.50	3.67	3.29	25		3.00	4.00	3.88	4.02	0.840
	Normal	20	83.3%					23	92.0%					0.318
	Mild	2	8.3%					0	0.0%					
	Moderate	2	8.3%					1	4.0%					
	Severe	0						0						

T-tests for continuous variables and chi-square tests for categorical variables*VAS* Visual Analogue Scale, *KHQ* King’s Health Questionnaire, *ICIQ-UI SF* International Consultation on Incontinence Questionnaire – urinary incontinence short form, *GSE-UI* Geriatric Self-Efficacy index for urinary incontinence, *HADS* Hospital Anxiety and Depression Scale

**Table 3:** Median, interquartile range (IQR), mean, standard deviation (SD) and p-values of outcome measurements at 12-week follow up

Variable	Dimensions	Intervention group						Control group						*p*-value
		*n*	%	Median	IQR	Mean	SD	*n*	%	Median	IQR	Mean	SD	
EQ-5D-5L	Mobility													0.478
	No/slight problem	21	87.5%					20	80.0%					
	Moderate/Severe/extreme problem	3	12.5%					5	20.0%					
	Selfcare													0.322
	No/slight problem	24	100.0%					24	96.0%					
	Moderate/Severe/extreme problem	0	0.0%					1	4.0%					
	Usual activities													0.950
	No/slight problem	20	83.3%					21	84.0%					
	Moderate/Severe/extreme problem	4	16.7%					4	16.0%					
	Pain & discomfort													0.682
	No/slight problem	17	70.8%					19	76.0%					
	Moderate/Severe/extreme problem	7	29.2%					6	24.0%					
	Anxiety & depression													0.957
	No/slight problem	21	87.5%					22	88.0%					
	Moderate/Severe/extreme problem	3	12.5%					3	12.0%					
	Index	24		0.92	0.26	0.86	0.16	25		0.88	0.16	0.83	0.19	0.471
	VAS	24		80.00	20.00	78.54	17.60	25		75.00	15.00	73.00	14.93	0.240
KHQ	Part I													
	General health perception (0-100)	24		25.00	12.50	30.21	23.29	25		25.00	25.00	31.00	20.77	0.900
	Incontinence impact (0-100)	24		33.33	33.33	45.83	21.56	25		33.33	33.33	49.33	25.68	0.609
	Part II													
	Role limitations (0-100)	24		16.67	33.33	20.14	23.04	25		33.33	33.33	30.67	23.90	0.123
	Physical limitations (0-100)	24		16.67	33.33	22.92	22.42	25		33.33	33.33	32.67	24.76	0.156
	Social limitations (0-100)	24		0.00	5.56	5.56	10.87	25		0.00	11.11	8.22	20.98	0.581
	Personal relationships (0-100)	16		0.00	0.00	0.00	0.00	16		0.00	0.00	5.21	11.74	**0.086**
	Emotions (0-100)	24		11.11	22.22	12.50	14.40	25		11.11	22.22	16.44	20.06	0.435
	Sleep/energy (0-100)	24		16.67	33.33	27.08	31.01	25		33.33	33.33	37.33	31.28	0.255
	Severity measures (0-100)	24		25.00	16.67	26.39	17.66	25		33.33	25.00	37.33	20.99	**0.055**
	Part III													
	Symptom severity scale (0-30)	24		6.00	4.00	6.38	2.93	25		10.00	4.00	9.04	3.51	**0.006**
ICIQ-UI SF	Scale (0-21)	24		4.50	4.00	5.38	2.83	25		9.00	5.00	7.76	4.08	**0.022**
GSE-UI	Scale (0-120)	24		94.50	31.00	89.13	23.86	25		67.00	34.00	69.08	25.46	**0.007**
HADS	Anxiety scale (0-21)	24		5.00	6.00	5.13	4.59	25		7.00	5.00	7.80	4.65	**0.049**
	Normal	19	79.2%					16	64.0%					0.593
	Mild	2	8.3%					4	16.0%					
	Moderate	2	8.3%					2	8.0%					
	Severe	1	4.2%					3	12.0%					
	Depression scale (0-21)	24		2.00	3.50	3.08	3.28	25		3.00	3.00	3.44	3.92	0.732
	Normal	21	87.5%					22	88.0%					0.720
	Mild	2	8.3%					1	4.0%					
	Moderate	1	4.2%					1	4.0%					
	Severe	0	0.0%					0						

T-tests for continuous variables and chi-square tests for categorical variables *VAS* Visual Analogue Scale, *KHQ* King’s Health Questionnaire, *ICIQ-UI SF* International Consultation on Incontinence Questionnaire – urinary incontinence short form, *GSE-UI* Geriatric Self-Efficacy index for urinary incontinence, *HADS* Hospital Anxiety and Depression Scale

**Fig. 1:**
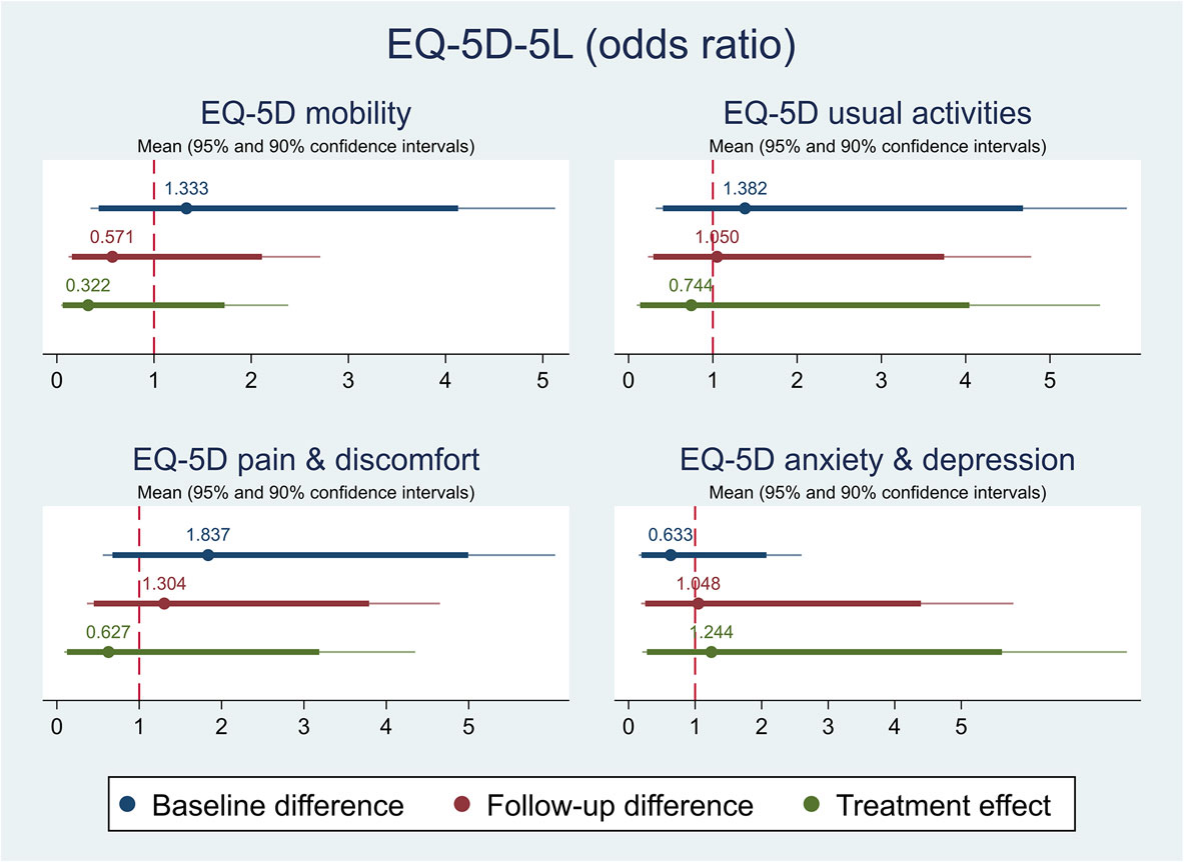
Mean (95 and 90% confidence intervals) of pre-post intervention differences and treatment effect for EQ-5D

**Fig. 2:**
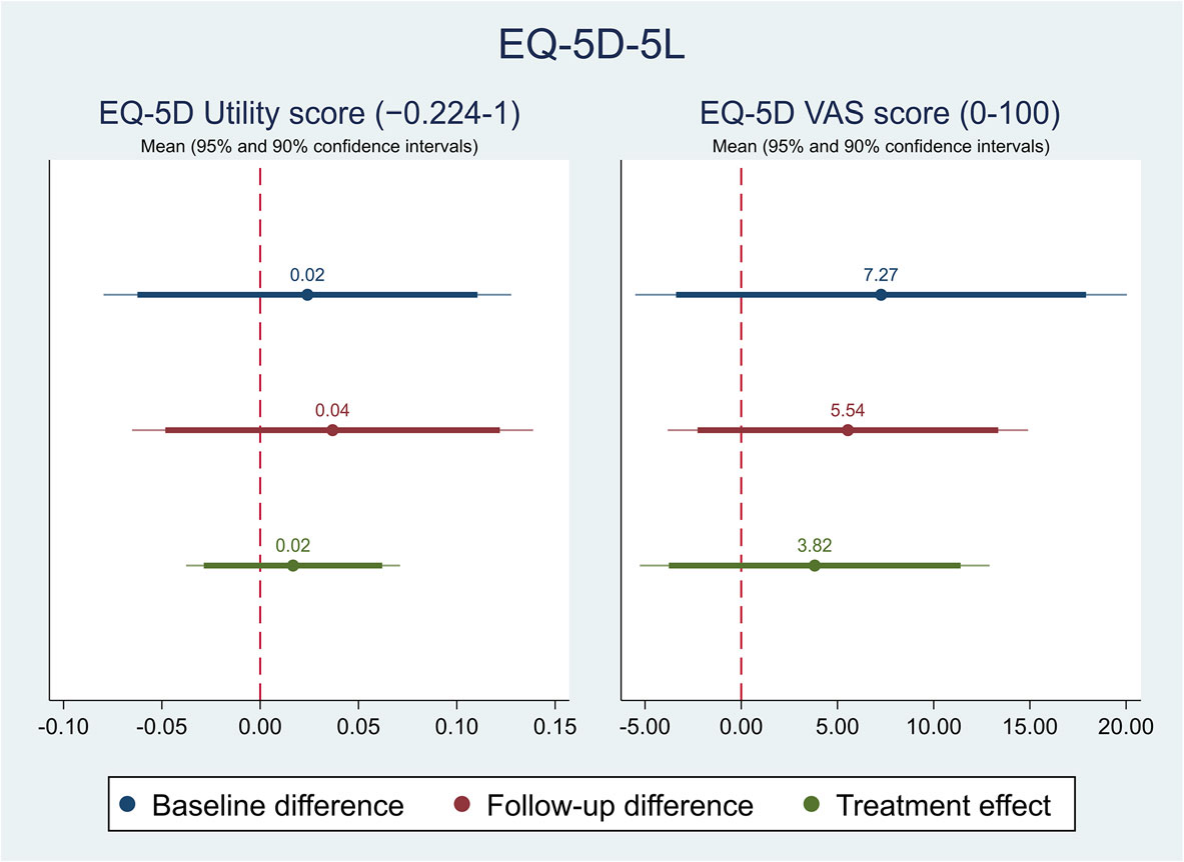
Mean (95 and 90% confidence intervals) of pre-post intervention differences and treatment effect for EQ-5D utility and VAS scores

**Fig. 3:**
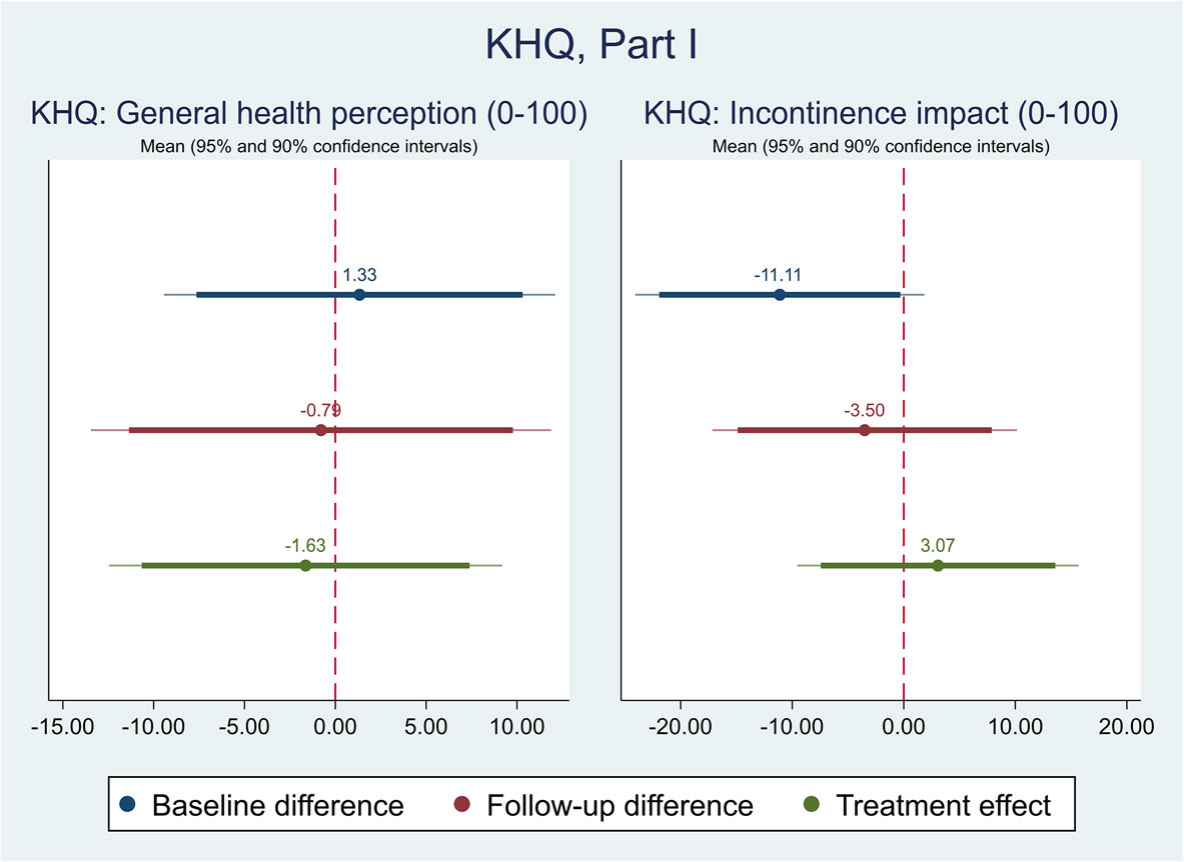
Mean (95 and 90% confidence intervals) of pre-post intervention differences and treatment effect for KHQ, Part I

**Fig. 4:**
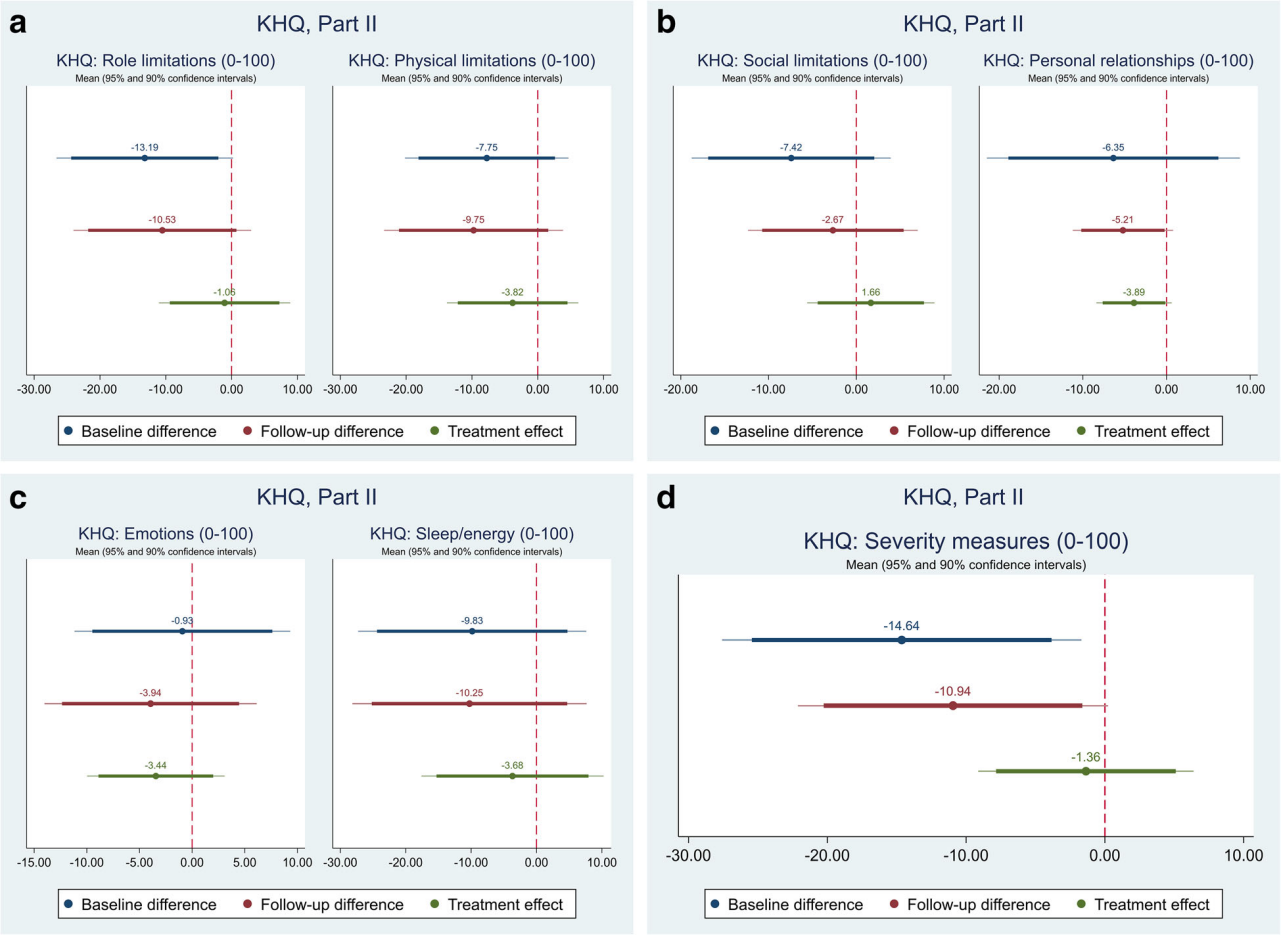
Mean (95 and 90% confidence intervals) of pre-post intervention differences and treatment effect for KHQ, Part II

**Fig. 5:**
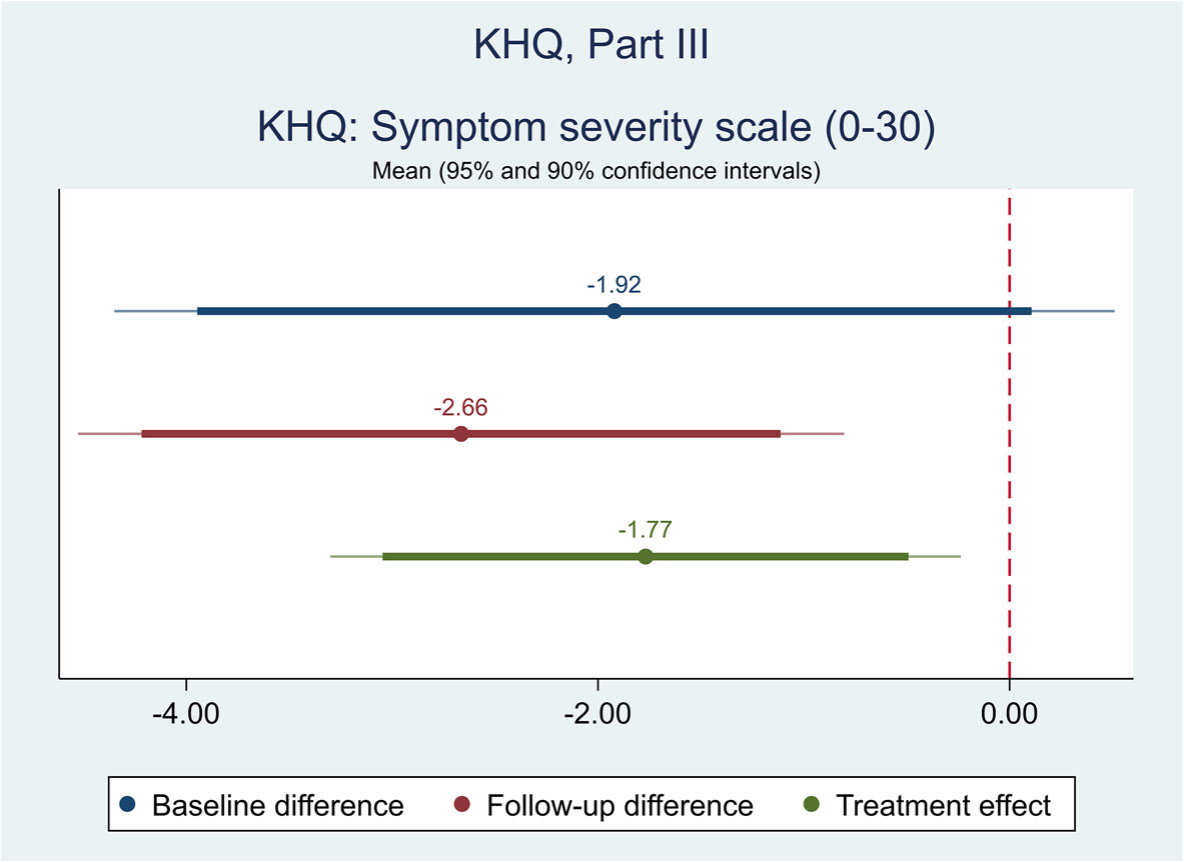
Mean (95 and 90% confidence intervals) of pre-post intervention differences and treatment effect for KHQ, Part III

**Fig. 6:**
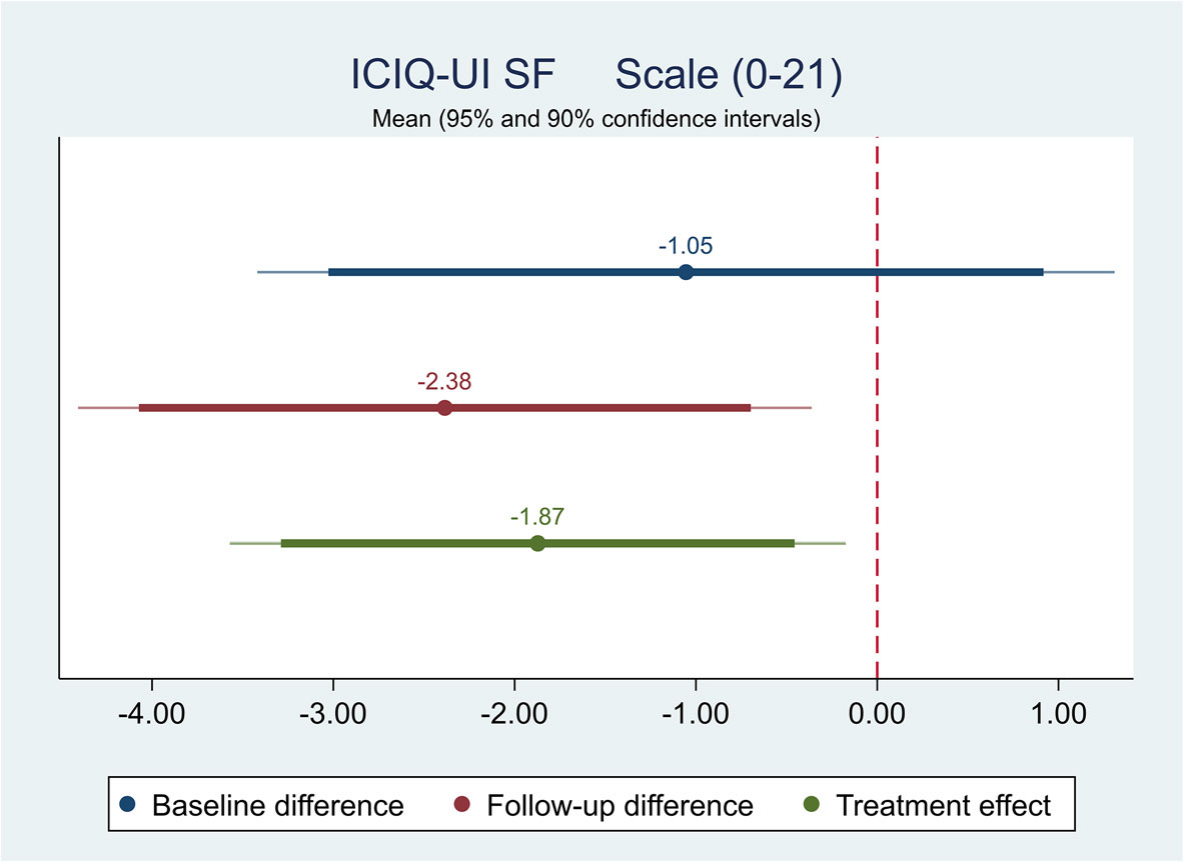
Mean (95 and 90% confidence intervals) of pre-post intervention differences and treatment effect for ICIQ-UI SF

**Fig. 7:**
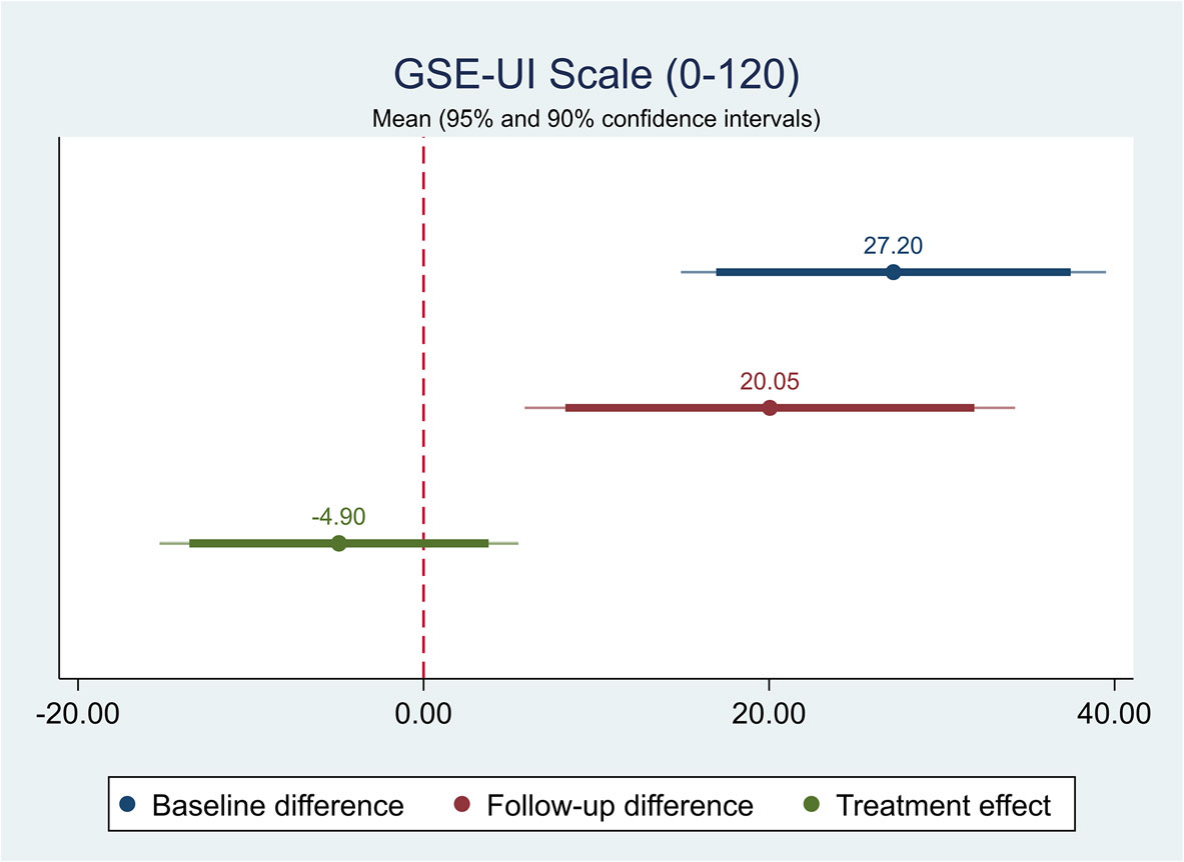
Mean (95 and 90% confidence intervals) of pre-post intervention differences and treatment effect for GSE-UI

**Fig. 8:**
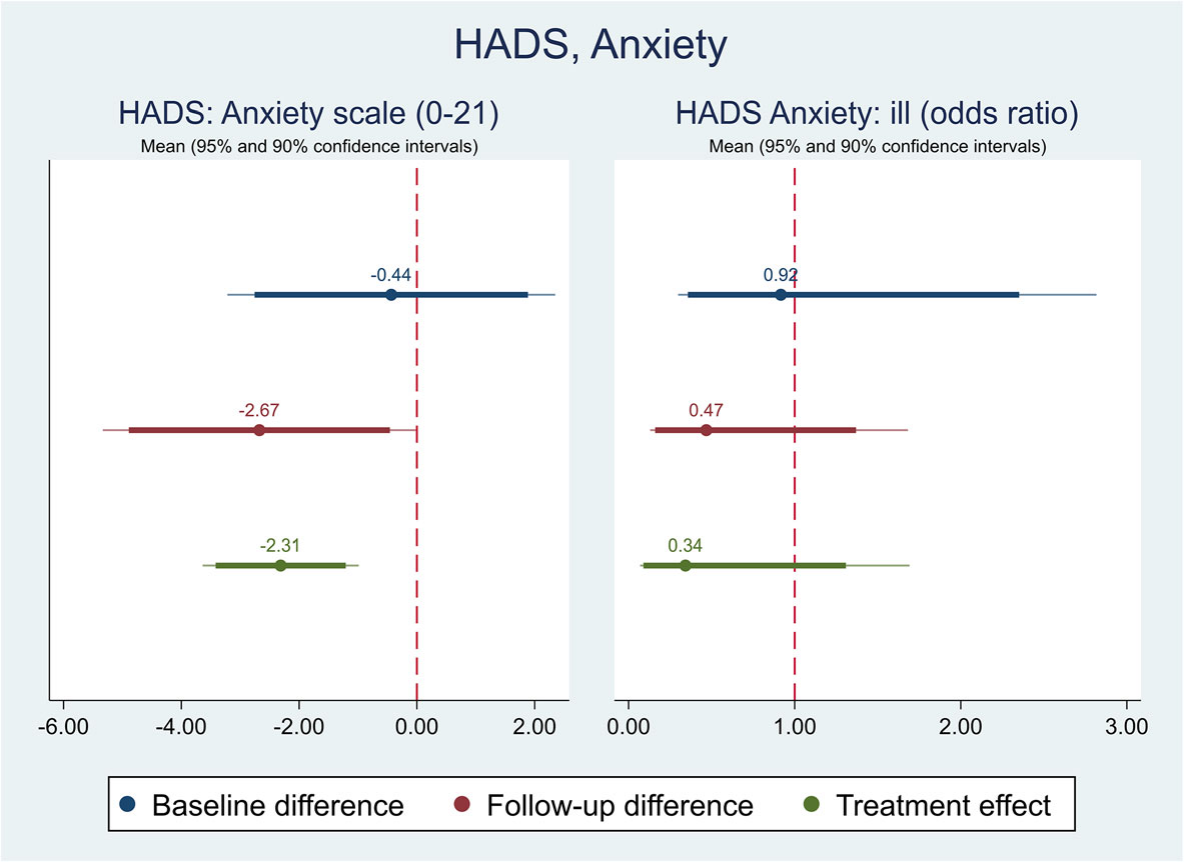
Mean (95 and 90% confidence intervals) of pre-post intervention differences and treatment effect for HADS, Anxiety

**Fig. 9:**
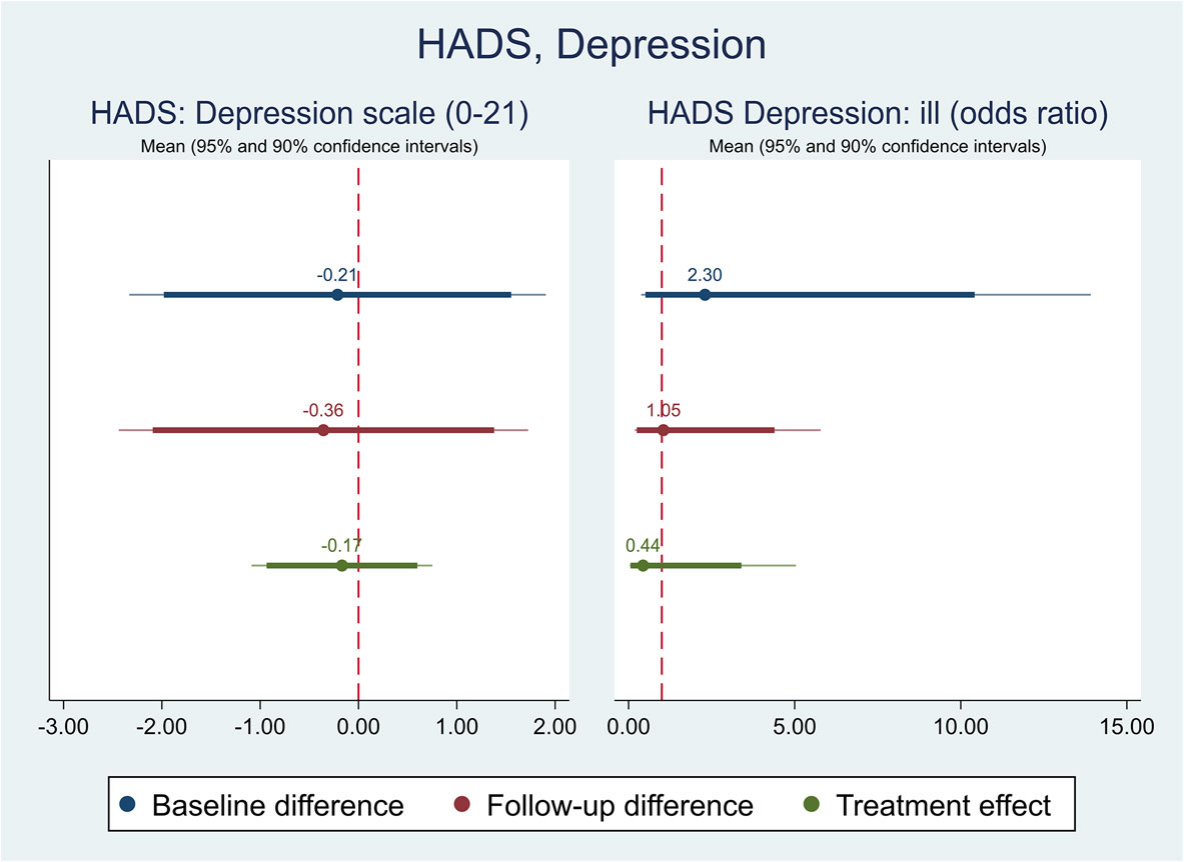
Mean (95 and 90% confidence intervals) of pre-post intervention differences and treatment effect for HADS, Anxiety

The effect of the self-management intervention on outcome measures between intervention and control groups were demonstrated in Table 4. The difference captured on each outcome measure was further shown in Figs. 1, 2, 3,4, 5, 6, 7, 8 and 9, respectively. Participants’ general quality of life were improved within both groups after the follow-up, however no significant difference was identified (see Figs. 1 and 2). Similarly, no difference was shown in UI specific quality of life (see Figs. 3 and 4), self-efficacy (see Fig. 7), or depression status (see Fig. 9) between groups. However, significant differences were detected in participants’ KHQ symptom severity (− 1.77, *p* = 0.025; also see Fig. 5), ICIQ-UI SF symptoms scale (− 1.87, *p* = 0.031; also see Fig. 6), and their anxiety status (− 2.31, *p* = 0.001; also see Fig. 8), respectively. A positive trend was also observed in the participants’ KHQ personal relationships (− 3.89, *p* = 0.088; also see Fig. 4).

**Table 4:** Mean, standard error (SE) and p-values of pre-post intervention differences and treatment effect

Variable	Dimensions	Baseline difference^a^			Follow-up difference^a^			Treatment effect^c^		
		OR^b^/Coef.	SE	*p-*value	OR^b^/Coef.	SE	*p-*value	OR^b^/Coef.	SE	*p-*value
EQ-5D-5L	Mobility									
	No/slight problem									
	Moderate/Severe/extreme problem	1.33	0.92	0.675	0.57	0.45	0.481	0.32	0.33	0.267
	Selfcare									
	No/slight problem									
	Moderate/Severe/extreme problem									
	Usual activities									
	No/slight problem									
	Moderate/Severe/extreme problem	1.38	1.02	0.663	1.05	0.81	0.950	0.74	0.77	0.774
	Pain & discomfort									
	No/slight problem									
	Moderate/Severe/extreme problem	1.84	1.12	0.317	1.30	0.85	0.683	0.63	0.62	0.637
	Anxiety & depression									
	No/slight problem									
	Moderate/Severe/extreme problem	0.63	0.46	0.526	1.05	0.91	0.957	1.24	1.14	0.811
	Index	0.02	0.05	0.643	0.04	0.05	0.471	0.02	0.03	0.540
	VAS	7.27	6.35	0.258	5.54	4.66	0.240	3.82	4.51	0.402
KHQ	Part I									
	General health perception (0-100)	1.33	5.35	0.804	-0.79	6.30	0.900	-1.63	5.38	0.763
	Incontinence impact (0-100)	-11.11	6.44	**0.091**	-3.50	6.79	0.609	3.07	6.26	0.626
	Part II									
	Role limitations (0-100)	-13.19	6.66	**0.054**	-10.53	6.71	0.123	-1.06	4.96	0.831
	Physical limitations (0-100)	-7.75	6.17	0.216	-9.75	6.76	0.156	-3.82	4.96	0.446
	Social limitations (0-100)	-7.42	5.64	0.195	-2.67	4.80	0.581	1.66	3.61	0.648
	Personal relationships (0-100)	-6.35	7.37	0.396	-5.21	2.93	**0.086**	-3.89	2.19	**0.088**
	Emotions (0-100)	-0.93	5.09	0.856	-3.94	5.01	0.435	-3.44	3.24	0.294
	Sleep/energy (0-100)	-9.83	8.67	0.263	-10.25	8.90	0.255	-3.68	6.92	0.598
	Severity measures (0-100)	-14.64	6.44	**0.028**	-10.94	5.55	**0.055**	-1.36	3.86	0.725
	Part III									
	Symptom severity scale (0-30)	-1.92	1.21	0.119	-2.67	0.93	**0.006**	-1.77	0.76	**0.025**
ICIQ-UI SF	Scale (0-21)	-1.06	1.18	0.374	-2.39	1.01	**0.022**	-1.87	0.84	**0.031**
GSE-UI	Scale (0-120)	27.20	6.11	**0.000**	20.05	7.05	**0.007**	-4.90	5.16	0.348
HADS	Anxiety scale (0-21)	-0.44	1.38	0.755	-2.68	1.32	**0.049**	-2.31	0.66	**0.001**
	Normal									
	Ill	0.92	0.53	0.879	0.47	0.31	0.245	0.34	0.28	0.188
	Depression scale (0-21)	-0.21	1.05	0.840	-0.36	1.03	0.732	-0.17	0.46	0.715
	Normal									
	Ill	2.30	2.11	0.364	1.05	0.91	0.957	0.44	0.54	0.505

*VAS* Visual Analogue Scale, *KHQ* King’s Health Questionnaire, *ICIQ-UI SF* International Consultation on Incontinence Questionnaire – urinary incontinence short form, *GSE-UI* Geriatric Self-Efficacy index for urinary incontinence, *HADS* Hospital Anxiety and Depression Scale^a^Difference=intervention-control; ^b^OR: odds ratio; ^c^For each outcome, analysis of covariance (ANCOVA) model with the baseline outcome was used to estimate the treatment effect

The relationship between the participants’ perceived improvement and the difference in outcomes within the intervention group was assessed using Spearman correlations (Table 5). There was a significant correlation shown between PGI-I and GSE-UI (*r*_*s*_= 0.43, *p* < 0.05). Mean change in GSE-UI for participants who responded “better” on the PGI-I were significantly higher than those who responded “no change” or “worse”.

**Table 5:** Correlation between PGI-I and changes in outcomes within intervention group

Mean change (SD)	PGI-I							Spearman's correlation
	Very much worse	Much worse	A little worse	No change	A little better	Much better	Very much better	
	0 (0%)	0 (0%)	4 (16.7)	3 (12.5)	9 (37.5)	7 (29.2)	1 (4.2)	
EQ-5D-5L								
Index	NA	NA	0.03 (0.10)	0.08 (0.01)	0.05 (0.09)	0.05 (0.14)	0.00 (.)	-0.05
VAS	NA	NA	2.50 (16.58)	8.33 (23.63)	13.89 (18.33)	2.86 (14.39)	-15.00 (.)	-0.04
KHQ								
Part I								
General health perception (0-100)	NA	NA	12.50 (43.30)	8.33 (14.43)	-8.33 (17.68)	-14.29 (13.36)	25.00 (.)	-0.25
Incontinence impact (0-100)	NA	NA	-8.33 (16.67)	0.00 (33.33)	7.41 (27.78)	-4.76 (12.60)	0.00 (.)	0.05
Part II								
Role limitations (0-100)	NA	NA	-8.33 (9.62)	5.56 (9.62)	-0.00 (18.63)	2.38 (17.82)	0.00 (.)	0.15
Physical limitations (0-100)	NA	NA	8.33 (28.87)	0.00 (16.67)	1.85 (15.47)	-4.76 (8.13)	-16.67 (.)	-0.19
Social limitations (0-100)	NA	NA	-5.56 (6.42)	0.00 (0.00)	-0.62 (14.28)	-7.94 (12.36)	0.00 (.)	-0.05
Personal relationships (0-100)	NA	NA	-16.67 (16.67)	0.00 (.)	0.00 (0.00)	-6.67 (14.91)	0.00 (.)	0.29
Emotions (0-100)	NA	NA	-11.11 (20.29)	0.00 (0.00)	-3.70 (9.62)	-6.35 (8.74)	22.22 (.)	0.08
Sleep/energy (0-100)	NA	NA	-4.17 (20.97)	-22.22 (9.62)	-7.41 (23.73)	-21.43 (20.89)	50.00 (.)	-0.02
Severity measures (0-100)	NA	NA	-12.50 (4.81)	-22.22 (17.35)	-3.70 (10.30)	-4.76 (13.49)	0.00 (.)	0.38*
Part III								
Symptom severity scale (0-30)	NA	NA	-1.25 (6.50)	0.00 (4.36)	-1.89 (3.92)	-2.43 (3.78)	0.00 (.)	-0.13
ICIQ-UI SF								
Scale (0-21)	NA	NA	-2.75 (2.22)	-1.33 (4.51)	-3.89 (3.66)	-3.86 (4.34)	-1.00 (.)	-0.02
GSE-UI								
Scale (0-120)	NA	NA	-15.00 (17.03)	0.67 (11.02)	8.78 (9.87)	10.71 (15.64)	4.00 (.)	0.43**
HADS								
Anxiety scale (0-21)	NA	NA	-1.75 (0.96)	-0.67 (1.53)	-1.11 (1.96)	-4.14 (3.34)	0.00 (.)	-0.22
Depression scale (0-21)	NA	NA	-1.00 (1.63)	0.33 (0.58)	-0.56 (1.88)	-0.86 (2.12)	0.00 (.)	-0.02

*PGI-I* Patient Global Impression: Improvement, *VAS* Visual Analogue Scale, *KHQ* King’s Health Questionnaire, *ICIQ-UI SF* International Consultation on Incontinence Questionnaire – urinary incontinence short form, *GSE-UI* Geriatric Self-Efficacy index for urinary incontinence, *HADS* Hospital Anxiety and Depression Scale**p < 0.05; *p < 0.1

### Qualitative findings

Individual interviews were undertaken with 15 participants from the intervention group at their homes. Participants ranged in age from 55 to 86 years. The majority were retired (11; 73%) and had suffered from UI for more than 5 years (11; 73%), but had received no support (13; 87%) from health services.

Each interview lasted between 15 and 48 min. A total of four themes were generated. Facilitators and barriers to the use of the self-management package followed by suggestions were also explored under each theme.

#### Raising awareness and gaining/refreshing knowledge

All participants found that this package had raised their attention to UI management as well as the resources and support available. Some participants learned new knowledge and management skills, while others found it useful to help refresh their memory. Positive comments were received on the fact that this package gathered both information and practical skills in a systematic and structured manner, accompanied by tips shared by women and health professionals.

*“Yeah, I found the information on how to do the pelvic floor muscle exercises really good, it describes exactly what you should do and how much you should do..” (W5)*

A few participants also expressed the need for more detailed information and tips to meet their needs. For instance, information and explanation were desired for biofeedback, vaginal cones, pads and how certain lifestyles impacted on UI symptoms and severity. In addition, some participants shared strategies and experiences of managing UI, including yoga, weight management programmes, and using reminders to practice PFME.

Half of the participants also identified the bladder diary as a potential barrier to the use of this book. For people who were in employment or regularly involved in outdoor activities, they felt that it was difficult and infeasible to measure intake of fluids and volume voided.

#### Feeling more confident and motivated to management UI

Participants generally felt more equipped and confident in managing their UI with resources and support supplied in the package.

*“…we can do this, at least we know where to contact, we can tell the doctors also but if it happens to somebody you can tell them and they can do this.”(W24)*

Participants also expressed their desire to motivate for self-management, however many of them did not practice as much as they would like to due to other commitments. Hence they suggested the inclusion of information and strategies on how to stay motivated and develop adherence.

*“I suspect a lot of people would read the book, and think, oh yeah, that’s a really good idea, I ought to be doing that, and then never, ever actually do it.”(W46)*

#### Being a useful and user-friendly package

All participants preferred the fact that this package was colour coded, making it easy to read and follow. They found the texts being easy to read and understand and the illustrations being appropriate, indicating that there was no need to book the support session. Some participants described benefit gained as a result of using this package.

*“The book was very well put together, and very clear, very easy to read, and it was very informative.” (W6)*

*“I’m still having the occasional accident, but now I’m sort of going oh well, I will get there. It’s not a major setback. But it’s not affecting my life like it used to.” (W5)*

Some of them preferred a smaller size of the package so they could carry in their handbags, whereas some believed that people tended to put it away if it was a small book. A few concerned about the phrase “older women” used in the title of the package and suggested to remove it or simply indicate “women over 55”.

#### Exploring dissemination strategies

All participants found the self-reported questionnaires in the feasibility study acceptable and considered them being necessary. Some appreciated being involved in this study. All participants recommended places where appropriate to distribute the package, including doctor’s surgeries, hospitals, pharmacies, workplaces, public libraries, local leisure centres, and other meeting places for women.

Some participants also expressed the need to design the package in multiple languages groups who may have limited access to support and resources. In addition to delivering the package to individuals, a couple of participants expressed their preference for a group to enable peer support.

## Discussion

This results of this study provide supporting evidence for the feasibility and acceptability of the self-management package as a complementary strategy for the management of UI in older women. This results of the feasibility RCT have confirmed that recruitment of women with UI from local community centres who were not actively engaged with health services is feasible and the drop-out rate is low. Completion and follow up of the questionnaires were successful. The findings of the acceptability study have suggested that the package may be a useful resource in improving women’s knowledge and practical skills for the management of UI. Women also expressed the need for such intervention for this condition and commented that questionnaires used to capture information were acceptable.

The feasibility RCT showed that women’s UI severity, symptoms and anxiety status were improved after the intervention for 3 months. This provides initial evidence that this self-management package has the capacity to reduce both physical and emotional impact of UI on women. There is an increasing interest in the self-management of UI consisting in both research and practice, however many interventions only focused on one particular skill or technique [6]. The UK Continence Society has recommended a support package for patients with UI as a minimum standard for continence care [41]. With no evidence-based package being available, this study developed the intervention consisting of multiple components and also demonstrated the acceptability of the intervention and potential benefit on women’s physical and psychological health. Therefore, future studies are needed to evaluate the intervention in other settings in a larger multicentre trial in order to gain robust evidence.

Although the difference in women’s quality of life or self-efficacy was not detected, women described that they felt more confident and motivated to self-manage their condition and some of them gained improvement after using the package. Qualitative interviews further supported these findings and participants reported that the package was informative and useful in raising their awareness. A significant correlation was observed between women’s self-efficacy and their subjective perception of improvement. This suggests that women who gained a higher level of self-efficacy were likely to experience an improved subjective perception of their UI condition. Therefore, individual’s self-efficacy level needs to be assessed as a way to stratify participants before randomisation process. For those with extremely low self-efficacy, interventions with a purpose to build their confidence may be needed before providing self-management package.

Existing literature has shown modest benefit of self-management interventions in older women with UI. Compared to pharmacological therapies alone, nonpharmacological interventions, such as behavioural therapy either alone or combined with other intervention, are more effective in achieving cure or improvement both stress and urge UI [42]. Clinical effectiveness of self-management of UI has also been with respect to symptoms, UI severity, quality of life and perceptions of improvement when being delivered in group format with effects maintained (12 months [43]. Similarly, this study has observed improvement in symptoms and severity with the self-management package, and effects on psychological health. This package appears feasible to modify women’s quality of life living with UI. Further studies may be needed to investigate the effectiveness of self-management interventions on both physical and psychosocial outcomes in women with UI, at both short and long term.

### Strengths and limitations

The intervention was co-developed with key stakeholders including women with UI and health professionals. The feasibility and acceptability of the intervention were evaluated with women who were not actively engaged with clinical services for UI. Potential positive effects were observed in women’s UI symptoms and their psychological status, which were further supported by the women’s experiences. However, careful interpretation is needed. The study was not allocation concealed and the sample size was not sufficiently powered to make robust inferences about the effectiveness of the intervention. Further studies are needed with a large sample size in multiple clinical settings to evaluate the effectiveness of this package. There is a possibility of bias associated with the self-reported measures. Objective information such as pad test or measure of PFM strength may be needed. It is worth noting that pad tests are not recommended in the routine assessment of women with UI by the NICE [44]. Also, women may be less in favour of the invasiveness of the measures such as vaginal dynamometer.

## Conclusions

The self-management package consisting of multifaceted behavioural techniques appears feasible to improve UI symptoms, severity and anxiety status in older women living with UI. Women also found it useful for increasing their awareness, motivation and knowledge of UI management. This study suggested that this package has the potential to be implemented in routine practice with further evaluation of effectiveness in clinical settings.

## Data Availability

The datasets used and/or analysed during the current study are available from the corresponding author on reasonable request.

## Abbreviations

MRC: Medical Research Council
PFME: Pelvic floor muscle exercises
RCT: Randomised controlled trial
UI: Urinary incontinence
